# Gait-initiation onset estimation during sit-to-walk: Recommended methods suitable for healthy individuals and ambulatory community-dwelling stroke survivors

**DOI:** 10.1371/journal.pone.0217563

**Published:** 2019-05-29

**Authors:** Gareth D. Jones, Darren C. James, Michael Thacker, Rhian Perry, David A. Green

**Affiliations:** 1 Centre for Human and Applied Physiological Sciences (CHAPS), King's College London, London, United Kingdom; 2 Physiotherapy Department, Guy’s & St. Thomas’ NHS Foundation Trust, London, United Kingdom; 3 Sport and Exercise Science Research Centre, School of Applied Sciences, London South Bank University, London, United Kingdom; University of Rochester, UNITED STATES

## Abstract

**Background:**

Gait-initiation onset (GI-onset) during sit-to-walk (STW) is commonly defined by mediolateral ground-reaction-force (xGRF) rising and crossing a threshold pre-determined from sit-to-stand peak xGRF. However, after stroke this method [xGRFthresh] lacks validity due to impaired STW performance. Instead, methodologies based upon instance of swing-limb maximum-vertical-GRF [vGRFmaxSWING], maximum-xGRF [xGRFmax], and swing-limb heel-off [firstHEELoff] can be applied, although their validity is unclear. Therefore, we determined these methodologies’ validity by revealing the shortest transition-time (seat-off–GI-onset), their utility in routinely estimating GI-onset, and whether they exhibited satisfactory intra-subject reliability.

**Methods:**

Twenty community-dwelling stroke (60 (SD 14) years), and twenty-one age-matched healthy volunteers (63 (13) years) performed 5 standardised STW trials with 2 force-plates and optical motion-tracking. Transition-time differences across-methods were assessed using Friedman tests with post-hoc pairwise-comparisons. Within-method single-measure intra-subject reliability was determined using ICC_3,1_ and standard errors of measurement (SEMs).

**Results:**

In the healthy group, median xGRFthresh transition-time was significantly shorter than xGRFmax (0.183s). In both the healthy and stroke groups, xGRFthresh transition-times (0.027s, 0.695s respectively) and vGRFmaxSWING (0.080s, 0.522s) were significantly shorter than firstHEELoff (0.293s, 1.085s) (*p*<0.001 in all cases). GI-onset failed to be estimated in 48% of stroke trials using xGRFthresh. Intra-subject variability was relatively high but was comparable across all estimation methods.

**Conclusion:**

The firstHEELoff method yielded significantly longer transition-times. The xGRFthresh method failed to routinely produce an estimation of GI-onset estimation. Thus, with all methods exhibiting low, yet comparable intra-subject repeatability, averaged xGRFmax or vGRFmaxSWING repeated-measures are recommended to estimate GI-onset for both healthy and community-dwelling stroke individuals.

## Introduction

Stroke incidence is high in the UK with ~150,000 cases per year [1]. While inter-disciplinary stroke management has led to an 80% survival rate [2], nearly 40% of survivors require assistance with everyday activities [3]. These activities include transitional movements that are executed when initiating or arresting movement, for instance gait initiation (GI) or sit-to-stand (STS) [4, 5]. Stroke survivors find transitional movements particularly challenging, which contributes to an increased fall risk [6]. Therefore, effective post-stroke rehabilitation strategies that target transitional movements and reliable assessment metrics to track functionality are vital.

Sit-to-walk (STW) occurs as a transitional movement when the cardinal subtasks STS and GI merge during rising in the transition-phase between the seat-off and GI-onset movement events [7]. The transition-phase is prolonged when STS and GI separation is induced by older age (>65) [8], history of falls [9], stroke [7, 10], or as part of normal movement, for example when re-orientating prior to GI [11]. As a result, STW performance (in health and pathology) exists within a continuum between transitional and non-transitional movement.

A movement-phase duration can be reported accurately within a continuum of task performance if the movement events delineating it are classified consistently. In fact, standardised movement event nomenclature has been adopted for STS [12], and for GI from quiet-standing [13]. In contrast, whilst a nomenclature for healthy STW has been proposed based on that for STS [14], it has only been applied partially in practice due to difficulty in defining STW GI-onset via critical magnitudes of mediolateral ground-reaction-forces in pathological states. As a result, alternative GI-onset classifications have been utilised, for example the instance of first heel-off in community dwelling stroke patients [10].

When executed from quiet-standing, GI can be reduced to two phases both characterised by centre-of-pressure (CoP) displacements. GI-onset represents the start of a postural anticipation-phase before movement occurs at first heel-off (HO1), which itself is indicative of the start of a dynamic execution-phase [15]. The anticipation-phase is characterised by posterolateral CoP displacement towards the swing limb induced by neuromuscular activation [16] required to accelerate the passive whole-body-centre-of-mass (BCoM) forwards [17]. Quantifying CoP displacement onset is therefore considered a direct method of determining anticipation-phase GI-onset, and has been successfully utilised in healthy and pathological groups undertaking GI from quiet-standing [13, 18–20]. In contrast, whilst a modest GI anticipatory-phase has been reported in STW [21, 22], determining CoP displacement onset is challenging because GI is superimposed on a pre-existent and dynamic seat-off event [14].

An alternative approach in STW trials is to determine an appropriate mediolateral ground-reaction-force (xGRF, in a direction towards the stance limb) threshold—normalised to body weight (BW)—that if breached represents GI-onset as the swing limb is loaded (xGRFthresh method). As lower overall peak xGRFs are generated during rising in STS (~4.4%BW) compared to STW [~7.9%BW; 14], population threshold values (mean +2SDs) are based on the same subject’s STS peak xGRF, which is inconvenient and potentially inaccurate. In fact, the xGRFthresh method is not used clinically and was reported to be inappropriate in over half of subjects in a study testing Parkinson’s Disease patients, presumably due to a failure of xGRF to cross the pre-determined threshold, although the actual reason was not reported [23].

In contrast, GI-onset estimation methods using peak values such as maximum swing-limb vertical GRF (vGRFmaxSWING) have been employed in healthy [24, 25] and stroke subjects [7, 26] as indirect estimates of GI-onset. However, because of its dependence on vertical GRFs, it is possible that vGRFmaxSWING might place GI-onset later in the anticipation-phase compared to the xGRFthresh method, which by virtue of reflecting horizontal GRF is more strongly associated with the postural component of GI. Similarly, kinematic events such as HO1 (firstHEELoff), which have been utilised in healthy young adults [27], older adults [22], Parkinson’s disease [23], and stroke subjects [10], tend to place GI-onset even later around the beginning of the dynamic component of GI.

An alternative methodology to estimate GI-onset in STW, without requiring prior cohort threshold calculations is the use of the maximum xGRF directed toward the stance limb (xGRFmax). However, in neither healthy or pathological individuals has this methodology been evaluated in comparison with xGRFthresh [14], vGRFmaxSWING [7], or firstHEELoff [23].

Thus, our aim was to determine which of xGRFthresh, vGRFmaxSWING, xGRFmax, or firstHEELoff methodologies are optimal in estimating GI-onset in both healthy individuals and community-dwelling ambulatory stroke survivors based on: criterion 1: validity (shortest transition-phase time thereby placing close to seat-off and thus the anticipatory phase of GI); criterion 2: utility (ability to generate a plausible GI transition-phase time from every STW trial); and criterion 3: reliability (satisfactory intra-subject reliability).

## Methods

### Participants

Twenty community-dwelling ambulatory individuals with stroke (7 women; 13 men; mean age: 60 (SD 14) years; height: 1.6 (0.09) m; mass: 77.28 (16.11) kg) and twenty-one age and gender-matched healthy individuals (7 women; 14 men; age: 63 (13) years; height: 1.71 (0.54) m; mass: 70.03 (8.67) kg) volunteered and provided written informed consent to participate in the study that received UK Health Research Authority approval (IRAS project ID: 200113). Individuals with stroke were invited to participate if they were aged over 18 years, community-dwelling, presented with a supra-tentorial or infra-tentorial lesion associated with an ischaemic or haemorrhagic stroke, or possessed multiple stroke lesions at least 3 months prior to recruitment that resulted in hemiparesis involving the lower limb (<15; Rivermead Mobility Index [28]), but able to ambulate 10 m safely indoors without a walking aid. They were excluded if they experienced severe exercise-induced dizziness, an Abbreviated Mental Test Score (AMTS [29] ≤7/10 [30]), were unable to rise from a chair without use of arms, or had history of any injury/pathology that further impaired ambulation. There were 13 subjects that presented with right, and 7 with left hemispheric pathology; of which 15 were ischaemic and 5 were haemorrhagic. Median time since stroke prior to the study was 8 (IQR 5–25) months; Abbreviated Mental test Score (AMT; maximum score 10) was 9 (8–9); Nottingham Extended Activities of Daily Living Score (NEADLS [31], maximum score 22) was 15 (12–19); and Rivermead Mobility Index (RMI, maximum score 15) was 12 (10–13). Healthy subjects were asked to complete an established gait-laboratory health-screen questionnaire tool. Respondents declared their health status using the tool based on their recall of any healthcare utilisation over the previous year, prescribed medications, history of musculoskeletal or vestibular pathology, current pregnancy, or unstable medical/mental health conditions. Subjects were excluded if on discussion with the researcher it was agreed any declared conditions/treatments represented a risk if they participated in the experiment.

Between-group differences in subject characteristics were assessed using independent sample student’s *t*-tests for continuous data, and chi-squared test for homogeneity as a test of proportions for dichotomous nominal data. There were no significant differences except that individuals with stroke walked with a significantly slower 4 m gait velocity (0.58 (0.28) m.s^-1^) than healthy subjects (1.14 (0.14) m.s^-1^; *t*(21.588) = 7.364, *p*<0.001) whose velocities were within published normative (according to age and gender) ranges [32].

### Experimental procedure

**Fig 1:**
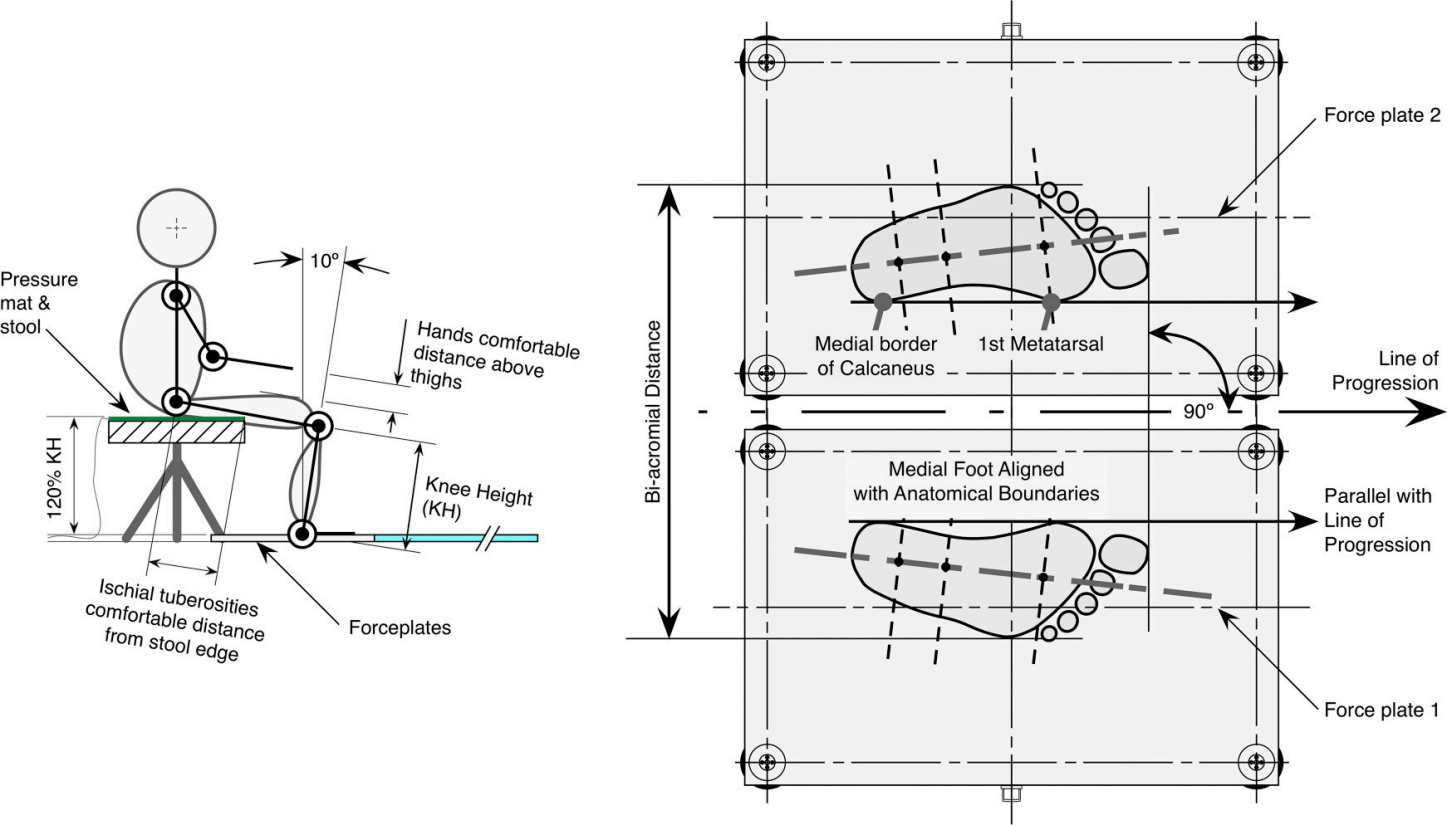
Standardised starting position. Subjects sat on an instrumented stool at 120% knee height (KH) with ischial tuberosities at a comfortable distance from the front edge, ankles 10° degrees in dorsiflexion, hands initially placed at a comfortable distance above thighs to avoid body marker obstruction (left schematic), and feet at shoulder width apart orientated forward (right schematic).

After familiarisation in the gait laboratory, subjects were asked to perform STW and STS (in order to derive thresholds for the xGRFthresh method) on 5 occasions each, in a randomised order during one measurement session. On each occasion subjects followed a novel low-risk protocol, the details of which are published elsewhere [33]. In brief, subjects rose from an instrumented (300 mm diameter pressure-mat, Arun Electronics Ltd, Sussex, UK) stool set at 120% knee height, with their feet in a standardised position upon 2 force plates (9281e; Kistler Instruments Ltd., Hook, Hants, UK) with hands initially placed at a comfortable distance above thighs to avoid body marker obstruction (Fig 1). Subjects were instructed in all trials, upon illumination of a light signal, to stand and walk forward (having led with their affected (stroke) or non-dominant (healthy) leg) ~5m along a walkway at a comfortable pace, stop and turn off the light using a switch. Subjects were instructed to move their arms naturally upon illumination of the light. In the STS trials, subjects commenced walking having paused once upright.

As part of a wider study, a 3D whole-body marker set was used, which was defined by placing 40 reflective markers (Qualysis AB, Gothenburg, Sweden) on skin overlying anatomical landmarks. Body segments were tracked using an additional 31 markers mounted in accordance with a six degrees-of-freedom marker-set [33]. Kinematic data were acquired using 10 infrared cameras (Oqus-3, Qualysis AB, Gothenburg, Sweden) sampled at 60Hz and synchronised with the analogue output from the force plates and seat-mat (1020Hz).

### Data analysis

Raw marker trajectories and analogue data were imported into Visual3D software (C-Motion Inc., USA). Kinematic and kinetic data were low pass filtered with a 10 Hz and 25 Hz 4th order low-pass Butterworth filter, respectively [33]. The pressure-mat analogue signal was filtered using 25-point window averaging in order to reproducibly determine seat-off.

To determine transition-phase time using the xGRFthresh method, the xGRF threshold value was calculated separately for the healthy and stroke groups and expressed as a percent of an individual’s bodyweight (%BW). The absolute sample mean peak xGRF from the summation of the two force plates during rising (movement-onset to upright) in STS was 2.0 (SD 0.6) and 2.6 (1.0) %BW in the healthy and stroke groups, respectively. Upon confirmation that STS peak xGRF was lower than corresponding value attained during STW (5.9 (1.4) and 4.8 (1.8) %BW, respectively), xGRFthresh was determined by calculating the *mean_STS_* + 2*SD_STS_* for both the healthy and stroke groups, which if crossed during rising in STW, was taken to indicate GI-onset [14].

Movement was analysed between events identifying the start of rising (movement-onset) and the end of GI at the first toe-off (TO1). Other events and parameters used to define data for analyses are summarised in Table 1.

**Table 1:** Movement events and GI-onset method definitions.

Event or Parameter	Definition		
Movement Onset	Instance determined when BCoM forward velocity signal (m.s^-1^) increases for >8 frames (>133ms) beyond the mean+3SD BCoM vertical velocity during 1s of quiet-sitting displacement prior to light-on		
Seat-Off	Instance determined as the point at which the seat-mat analogue channel voltage signal (v) drops below the mean-3SD baseline voltage for >8 frames (>133ms) of 1s quiet-sitting		
Upright	Instance of initial peak vertical (z-component) BCoM displacement signal (m) occurring between Seat-Off and 1^st^ Toe-Off events		
GI-Onset Estimation Method	1.	Mediolateral GRF Threshold (xGRFthresh)	Instance when summated force plates mediolateral (x-component, away from swing limb) GRF signal (%BW) breaches the group xGRF Threshold
	2.	Max Vertical GRF Swing (vGRFmaxSWING)	Instance of maximum swing-limb force plate vertical (z-component) GRF signal (N) occurring between Movement-Onset and 1^st^ Heel-Off events
	3.	Max Mediolateral GRF (xGRFmax)	Instance of local maximum summated mediolateral (x-component, toward the stance limb) GRF signal (N) occurring between Movement-Onset and 1^st^ Heel-Off event
	4.	1^st^ Heel-Off (firstHEELoff)	Instance when swing limb calcaneal marker vertical velocity signal (m.s^-1^) breaches >0.0 for ≥8frames (133ms) after Seat-Off event
1^st^ Toe-Off (TO1)	Instance when swing limb force plate vertical (z-component) GRF signal (N) drops <20N for >8 frames (133ms) occurring after Seat-Off event		

AP–anteroposterior; BCoM–whole body centre-of-mass; BW–Bodyweight (N)

GRF–ground reaction force; SD–standard deviation

GI-onset times were estimated for each STW trial across the 4 estimation methods, from which seat-off times were subtracted to derive transition-phase times. Resultant transition-phase times were averaged over all available trials for each subject. None of the averaged transition-phase data in the stroke group were normally distributed irrespective of estimation methods (Shapiro-Wilks test). Therefore, Mann Whitney U Tests were performed to determine whether there were differences in median transition-phase time between stroke and healthy groups for each GI-onset estimation method. Friedman tests were used to determine whether there were differences in STW transition-phase times across all GI-onset estimation methods, for the healthy and stroke groups independently with (Bonferroni corrected) pairwise comparisons (SPSS v24, IBM Corp, Armonk, NY, USA) to determine whether any method yielded significantly shorter transition-time durations (criterion 1). The proportion of trials in which GI-onset was unable to be determined (criterion 2) are reported for each method.

Intra-subject transition times reliability (criterion 3) for each GI-onset method was determined by assessing absolute agreement between STW trials using a two-way mixed model ICC_3,1_ [34] with 95% confidence intervals (CI). Additionally, one-way analyses of variance (ANOVA) were conducted to calculate the within-subject variance (total within-subjects mean square (MS_w_) value). The standard error of measurement (SEM) is simply the square-root of MS_w_. It is typically reported that the 95%CI for a subject’s true score (T) can then be estimated as the observed score (S) ±1.96(SEM) [35]. The SEM can therefore reveal the difference between a subject's measurement and the true value that would be expected for 95% of observations [36]. For all statistical tests (SPSS v24, IBM Corp, Armonk, NY, USA) significance was assumed at *p*≤0.05. The full dataset is available from the LSBU Research Open repository at https://doi.org/10.18744/LSBU.002933.

## Results

### Missing data

All 21 subjects completed all five walking trials for both tasks in the healthy group (n = 105 STW and n = 105 STS trials in total). Of the 20 subjects in the stroke group, one was unable to complete three STS and one STW trial, and no data could be extracted for one STS and one STW trial in another subject due to technical issues with the motion capture system (n = 98 STW and n = 96 STS trials in total). All available data were included in the analyses. The ICC_3,1_ calculation for each GI-onset estimation method were predicated on all 5 STW trials being present; subject data with any missing trials were not included in ICC_3,1_ calculations.

### xGRFthresh threshold calculations

Calculations showed that threshold values were significantly lower in the healthy (3.1%BW), compared to the stroke group (4.5%BW) [*t*(39) = -2.360, *p* = 0.023].

### Validity (criterion 1)

**Fig 2:**
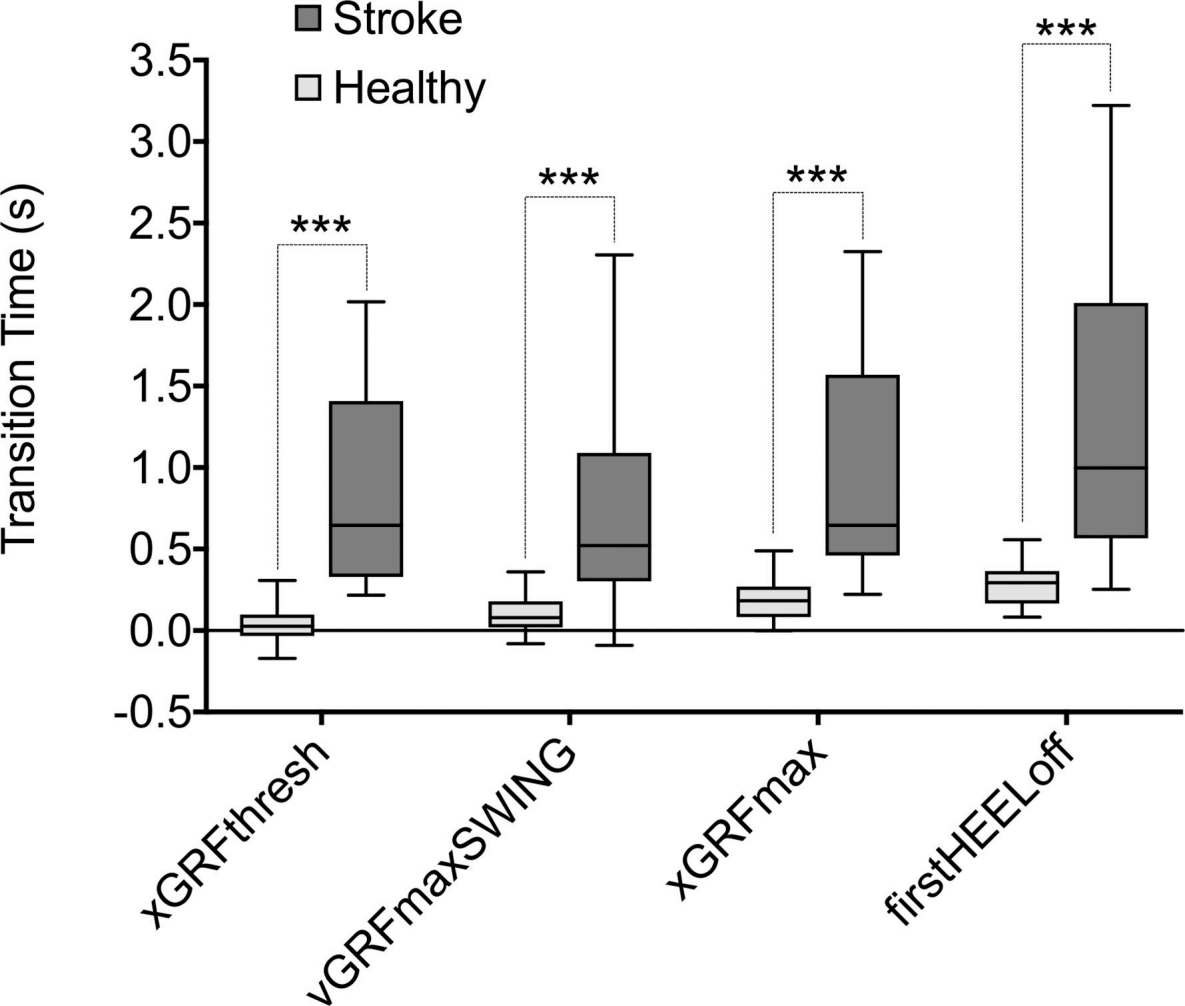
Between-group STW transition time box plots comparison for each GI-onset estimation method. Significant differences between-groups exist for all methods (Mann-Whitney U tests): *** indicates *p*<0.001.

Median transition-phase times were significantly shorter in the healthy group when compared to the stroke group for each GI-onset estimation method (xGRFthresh: 0.027 vs. 0.695s, *U* = 4, *z* = -5.028, *p*<0.001; vGRFmaxSWING: 0.080 vs. 0.522s, *U* = 43, *z* = -4.356, *p*<0.001; xGRFmax: 0.183 vs. 0.695s, *U* = 18, *z* = -5.008, *p*<0.001; firstHEELoff: 0.293 vs. 1.085s, *U* = 18.5, *z* = -4.995, *p*<0.001) (Fig 2).

Median transition-phase times were significantly different across the different GI-onset estimation methods in both the healthy [χ^2^(3) = 58.600; *p*<0.001] and stroke group [χ^2^(3) = 31.500; *p*<0.001].

**Fig 3:**
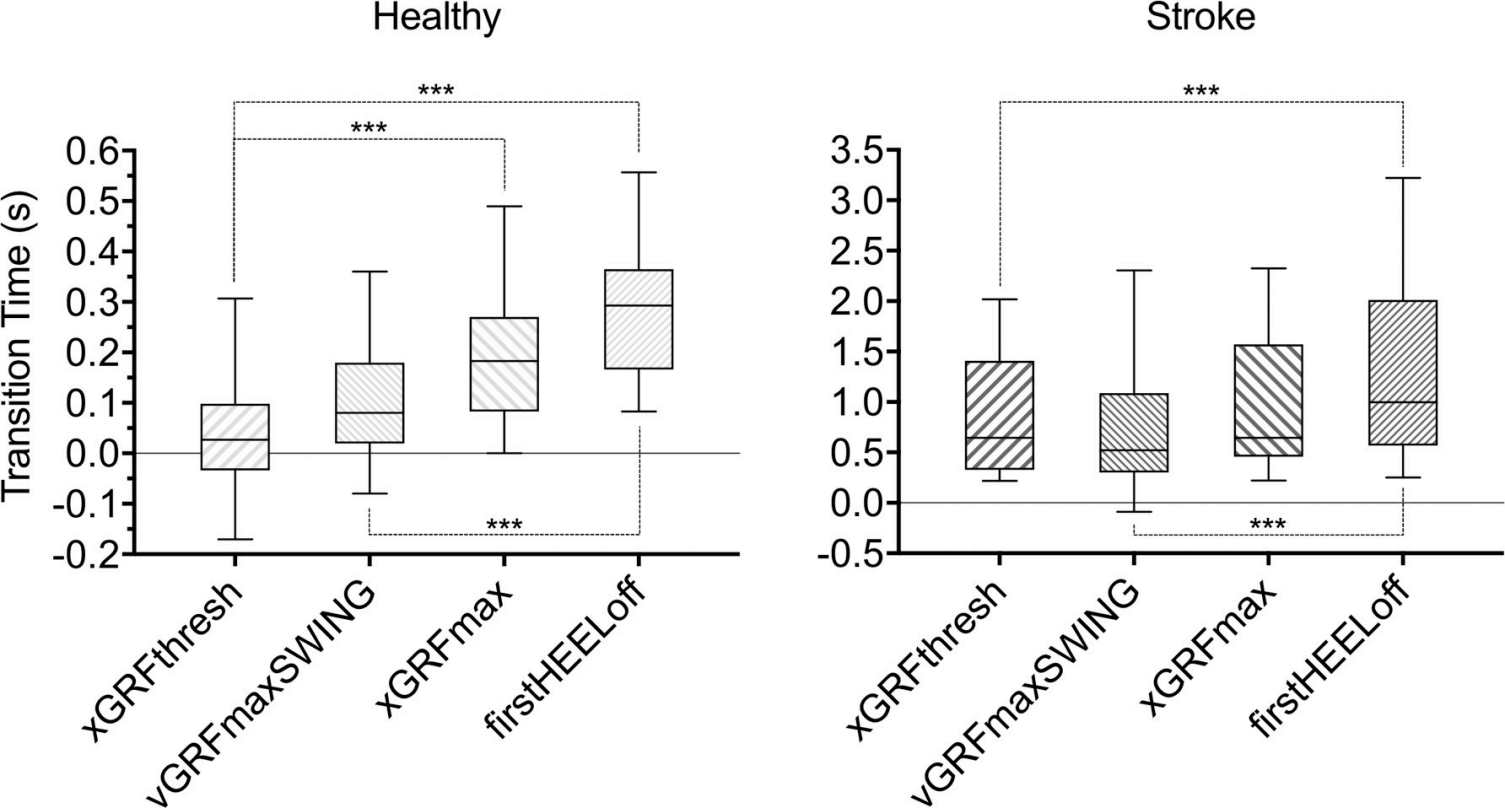
STW transition time box plot for healthy and stroke groups. Significant differences across GI-onset methods were observed within the healthy and stroke groups (Friedman test), dashed lines show statistically significant pairwise comparisons; *** represents statistically significant difference between GI-onset estimation methods at *p*<0.001 level.

In the healthy group xGRFthresh median (IQR) transition-phase time (0.027s (-0.033–0.098s)) was not significantly shorter than that derived by the vGRFmaxSWING (0.080s (0.020–0.180s)) method, but was significantly shorter than xGRFmax (0.183s (0.083–0.270s); *p*<0.001) and firstHEELoff (0.293s (0167–0.365s); *p*<0.001) methods. The vGRFmaxSWING method was also significantly shorter than the firstHEELoff method (*p*<0.001), but not xGRFmax Fig 3).

In the stroke group xGRFthresh median (IQR) transition-phase time (0.695s (0.329–1.508s)) was not significantly shorter than that derived by either the vGRFmaxSWING (0.522s (0.303–1.435s)) or xGRFmax (0.695s (0.460–1.588s)) methods, but was significantly shorter than the firstHEELoff method (1.085s (0.567–2.011s); *p*<0.001). The vGRFmaxSWING method yielded shorter transition-phase times that were also significantly shorter than the firstHEELoff (*p*<0.001), but not xGRFmax method.

### Utility (criterion 2)

**Fig 4:**
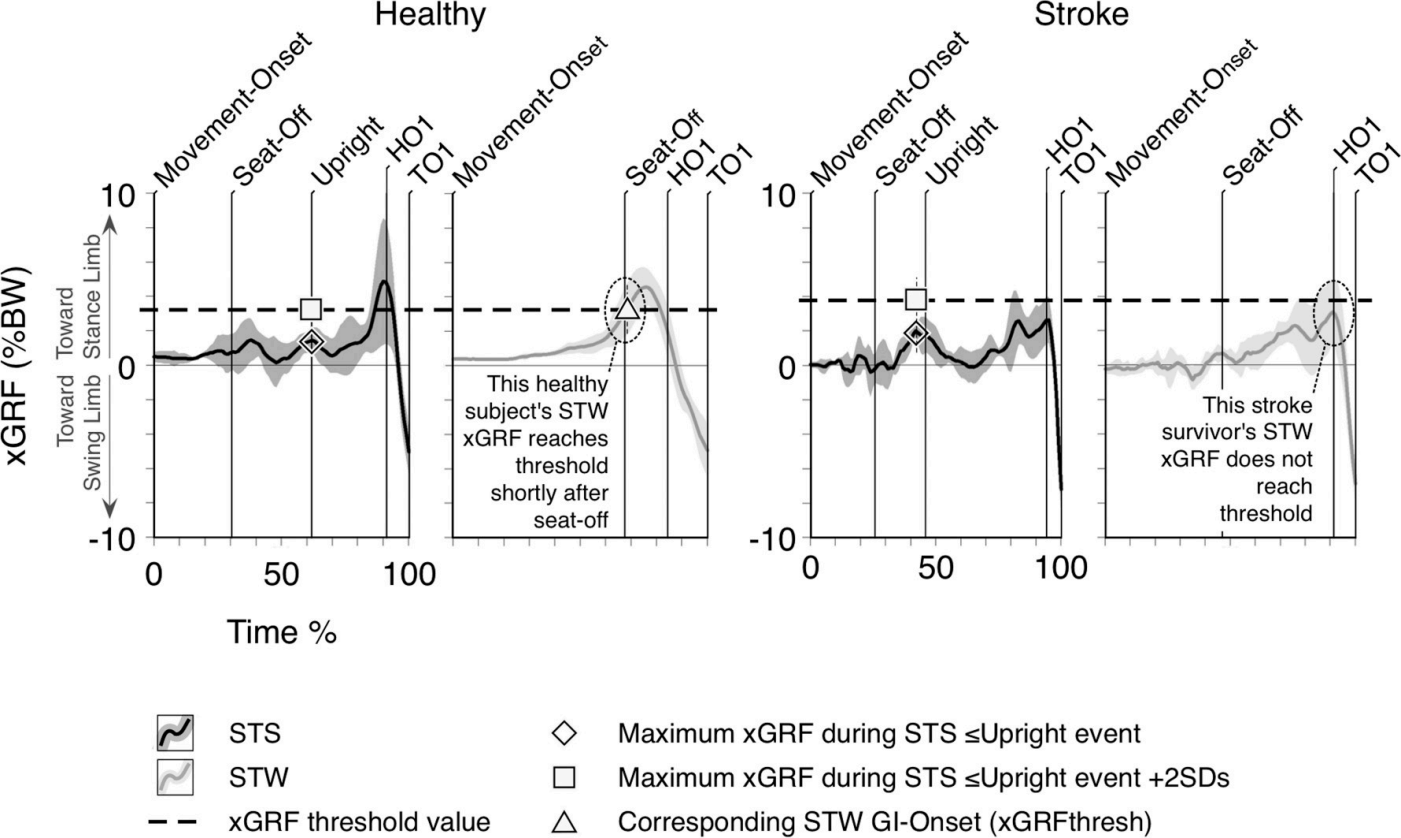
Typical mean (±1SD) xGRF profiles during 5 trials of STW and STS for a healthy, and a stroke survivor. Time axes represent percentage time from movement-onset to 1st toe-off (TO1). Darker lines represent STS and lighter lines STW. Mean time of event occurrences are shown as labelled vertical lines; mean maximal xGRF during rising in STS is labelled with +2SDs corresponding to the threshold values to estimate GI-onset for STW. In contrast to the healthy subject, the stroke survivor mean STW profile does not reach calculated xGRF threshold meaning GI-onset was undeterminable with xGRFthresh method.

GI-onset events, and hence transition-phase times, could be estimated from every STW trial undertaken using the vGRFmaxSWING, xGRFmax, and firstHEELoff methods in both groups. However, it was frequently impossible to determine GI-onset using the xGRFthresh method, particularly in the stroke group. In fact, peak xGRF failed to cross the subject-specific pre-determined threshold in 47 (out of a total of 98) stroke trials (48%) with no GI-onsets defined in any trial for five (out of 20) stroke subjects (Fig 4). In the healthy group, 6 (out of a total of 105) trials (6%) failed to cross the pre-determined xGRF threshold.

### Reliability (criterion 3)

Intra-subject reliability over the 5 STW trials was poor-to-moderate for all GI-onset estimation methods demonstrated by all ICCs <0.4 for the healthy, and ≤0.5 for stroke subjects (Table 2). The 95%CIs for each method, in both groups, overlapped.

**Table 2:** Transition time intra-subject variability for each estimation method. Results are shown per group as mixed effects model ICCs_3,1_ for absolute agreement with 95% confidence intervals (CI), and the standard error of measurement (SEM or within-subject standard deviation (*S*ω)).

		Healthy			Stroke	
Transition Time Method	*n*	ReliabilityICC_3,1_ (95%CI)	SEM	*n*	ReliabilityICC_3,1_ (95%CI)	SEM
xGRFthresh	19	0.328 (0.135–0.579)	0.151s	6	0.503 (0.170–0.878)	0.481s
vGRFmaxSWING	21	0.319 (0.135–0.557)	0.143s	18	0.446 (0.235–0.685)	0.651s
xGRFmax	21	0.371 (0.180–0.605)	0.158s	18	0.449 (0.241–0.687)	0.620s
firstHEELoff	21	0.356 (0.168–0.591)	0.164s	18	0.411 (0.203–0.658)	0.687s

ICC–Intraclass correlation coefficient, 2-way mixed-model with single measures

SEM–Standard error of measurement; CI–Confidence interval

*n*—represents the number of subjects presenting no missing data in all 5 STW trials

SEMs were large and comparable for all GI-onset estimation methods in the healthy group. SEMs were even greater in the stroke group and broadly comparable except for xGRFthresh, which was based on only 6 subjects due to missing data.

## Discussion

Our main findings are that estimating GI-onset using the xGRFthresh method results in short transition-phase durations in most healthy individuals and some community-dwelling stroke patients; therefore representing a valid method (criterion 1). However, its utility was poor (criterion 2) with GI-onset unable to be estimated in a high proportion of trials particularly in stroke. The firstHEELoff method lacked validity by generating significantly longer transition-phase times than xGRFthresh or vGRFmaxSWING in both groups. There was no significant difference in transition-phase time between the vGRFmaxSWING or xGRFmax methods, and intra-subject reliability (criterion 3) was poor-to-moderate for each estimation method in both groups.

The xGRFthresh method estimated GI-onset to be almost coincident with seat-off in healthy subjects (median (IQR): 0.027s (-0.033–0.098s)), consistent with previous work [14], and yielded the shortest transition-phase time. In the stroke group, where transition-phase times were significantly longer than in the healthy group irrespective of GI-onset estimation method, the xGRFthresh method did not yield the shortest transition-phase time but was nonetheless significantly shorter than the firstHEELoff method. As HO1 represents the end of the anticipatory-phase of GI in STW, GI-onset is placed within the anticipation-phase using the xGRFthresh method in both the healthy and stroke groups. Therefore, xGRFthresh represents an acceptable analogue for anticipatory-phase GI-onset in stroke, and the best analogue in health.

However, the xGRFthresh method failed to estimate GI-onset in a small proportion (6%) of trials in healthy subjects and in nearly half (48%) of the stroke groups’ STW trials. In fact, failure to estimate GI-onset using xGRFthresh has been reported previously as transitional movement dysfunction often renders individuals’ STW execution indistinguishable from STS due to an inability to perform STW without separation of the cardinal STS and GI sub-tasks [8–10]. The xGRFthesh method is therefore unlikely to detect GI-onset in trials where individuals do not perform STW fluidly [7]. Thus, while our data confirms the validity of the xGRFthresh method in healthy STW, its lack of utility suggests it is not appropriate when pathology or context limits fluidity between STS and GI. Unlike xGRFthresh, all other estimation methods used in this study satisfied the utility criterion by estimating GI-onset for all completed trials in both groups.

The firstHEELoff method yielded the longest transition-phase time, placing GI-onset significantly later than xGRFthresh and vGRFmaxSWING in both groups. Heel off represents the dynamic GI execution-phase onset in GI from quiet-standing [15] and STW [7], which occurs after the postural-phase. Therefore, it is unsurprising that it resulted in lengthy transition-phase times. Nonetheless, firstHEELoff has been used extensively to determine GI-onset in STW in healthy adults [22, 27] and pathology [10, 23], where the anticipation-phase of GI was deemed unimportant. However, our data confirms that for GI-onset definition across the continuum of STW performance, alternative and more appropriate methods exist.

The vGRFmaxSWING method estimated GI-onset within the anticipatory-phase in STW. This is evidenced by significantly shorter transition times, compared to the firstHEELoff method, in both healthy and stroke groups, and thereby satisfies our first two criteria (validity and utility). The vGRFmaxSWING method has been previously employed [24–26], not least to compare stroke and age-matched healthy individuals STW performance [7]. The authors of this study adopted a similar arms-unconstrained protocol as the present study, but with a lower fixed seat-height of 0.450m (compared to our 0.567 (0.035) m). They reported transition-phase times in stroke (0.49 (0.36) s) and healthy (0.14 (0.10) s) groups, which fall within the IQR reported here (stroke: 0.30–1.44; healthy: 0.02–0.18), suggesting that transition-phase time method is consistent across protocols. Thus, vGRFmaxSWING is a candidate methodology to estimate GI-onset estimates across the STW performance continuum from healthy to community-dwelling ambulatory stroke patients.

The xGRFmax method estimates GI-onset using the same kinetic signal as the xGRFthresh method and should therefore be associated with the anticipatory-phase of GI in STW. However, our data does not confirm that xGRFmax satisfies criterion 1 because transition-phase times were not significantly shorter than those using firstHEELoff. An advantage of xGRFmax on the other hand is that it requires only a single force plate, and future studies/clinical practice, whose prime purpose is not exclusively the identification of GI-onset, may benefit from this method.

Our third criterion was that GI-estimation methods should possess comparable estimated transition-time reliability. ICCs were poor-to-moderate throughout. However, as the 95% CIs overlapped around the low ICCs in both healthy and stoke groups, no single method can be determined as optimal in terms of reliability.

We observed less dispersion around the median in the healthy group for all GI-onset estimation methods which signifies between-subject homogeneity, where lower ICCs are mathematically possible because of their dependence on the magnitude of between-subject variation [35]. It is preferable therefore to avoid interpretation of low ICCs in isolation and instead consider them in conjunction with SEMs to determine an absolute index of reliability in the same units as the observations [37]. However, SEMs were large for all the GI-onset estimation methods confirming a large amount of movement variability.

In fact, poor-to-moderate agreement and a high degree of movement variability similar to ours in stroke (ICC_2,1_ = 0.54 (95%CI 0.28–0.82)) has been reported for transition-phase times in STW previously [7]. More variable movement has traditionally been assumed to be random error or noise associated with less stability confirmed by balance observations in older adults where increased movement variability is a predictor of falling [38].

This traditional view is questioned by the theory that highly variable movement is also observed in skilled movements. For example in a study of elite triple-jumpers, high movement variability was observed in the most skilled jumpers, and well as the least skilled [39]. Analogies of novices and experts balancing on balls [40] or walking a tightrope [41] help conceptualise that these data suggest different phenotypes of variability exist, which is in keeping with optimal variability theory [42]. According to the theory, skilled movement variability is non-random and complex signifying a presence of structured moment-to-moment movement variation representing physiologic adaptability to constantly changing demands [41]. In contrast, non-complex variability signifies non-structured and random movement representing a loss of adaptive capability. In pathology, lower complexity movement variability can be represented in two ways. Either by a high predictability of movement where variability is held and movement is rigid (e.g. limited coordination between joints after stroke [43]), or by low predictability where variability is withheld and movement is noisy (e.g. unsteady limb movement in cerebellar ataxia [44]).

In our data, we observed more favourable ICC_3,1_ values (less variability) in the stroke group, which as we have said, could be reflective of the mathematics of calculating ICCs. However, less variability in stroke could also be reflective of our stroke subjects reducing their risk of movement failure (falling) by withholding movement variability.

Overall, our reliability data confirm that STW analyses should not be based on individual readings. Instead they should always be based on the averaged repeated trials within a measurement session [36]. Previous work showed intra-session reliability was enhanced using the arithmetic mean of four repeated trials in standing balance [45, 46] and six trials in GI [47]. In practice, summary statistics based on the arithmetic mean of five trials has been used extensively to reduce random error from intra-individual variation in GI from quiet-standing [48–51] and STW [9, 14, 22, 23, 52], which is why we chose and recommend averaging of 5 trials. Future studies are required to determine clinically relevant minimal detectable changes based on test-retest averaged data between measurement sessions as this would be invaluable in clinical practice to monitor rehabilitation progress post-stroke.

## Conclusion

Our aim was to determine an optimal approach to estimate STW GI-onset suitable in both healthy and community-dwelling ambulatory stroke individuals from 4 different methods based on validity, utility and reliability criteria. The firstHEELoff method was the least valid by yielding significantly longer transition-times, thereby placing GI-onset at the end of the anticipation-phase of GI. The utility of the xGRFthresh method was poor because it failed to routinely estimate GI-onset, particularly in the stroke patients. In contrast, both the xGRFmax and vGRFmaxSWING methods were valid and presented with favourable utility using one and two force plates respectively. However, because single measure repeatability is poor-to-moderate for all estimation methods, averaging transition-phase times from multiple trials is required to mitigate high intra-subject variability. In conclusion, average repeated-measures using the xGRFmax or vGRFmaxSWING methods appear able to estimate GI-onset across the continuum of STW performance.
